# Relation between molecular electronic structure and nuclear spin-induced circular dichroism

**DOI:** 10.1038/srep46617

**Published:** 2017-04-24

**Authors:** Petr Štěpánek, Sonia Coriani, Dage Sundholm, Vasily A. Ovchinnikov, Juha Vaara

**Affiliations:** 1 NMR Research Unit, University of Oulu, PO Box 3000, FI-90014 Oulu, Finland; 2 Institute of Organic Chemistry and Biochemistry, AS CR, Flemingovo nam. 2, 166 10 Prague, Czech Republic; 3 DTU Chemistry - Department of Chemistry, Technical University of Denmark, 2800 Kgs. Lyngby, Denmark; 4 Department of Chemistry, University of Helsinki, FI-00014 Helsinki, Finland; 5 Emanuel Institute of Biochemical Physics RAS, Kosygin street 4, 119334 Moscow, Russia

## Abstract

The recently theoretically described nuclear spin-induced circular dichroism (NSCD) is a promising method for the optical detection of nuclear magnetization. NSCD involves both optical excitations of the molecule and hyperfine interactions and, thus, it offers a means to realize a spectroscopy with spatially localized, high-resolution information. To survey the factors relating the molecular and electronic structure to the NSCD signal, we theoretically investigate NSCD of twenty structures of the four most common nucleic acid bases (adenine, guanine, thymine, cytosine). The NSCD signal correlates with the spatial distribution of the excited states and couplings between them, reflecting changes in molecular structure and conformation. This constitutes a marked difference to the nuclear magnetic resonance (NMR) chemical shift, which only reflects the local molecular structure in the ground electronic state. The calculated NSCD spectra are rationalized by means of changes in the electronic density and by a sum-over-states approach, which allows to identify the contributions of the individual excited states. Two separate contributions to NSCD are identified and their physical origins and relative magnitudes are discussed. The results underline NSCD spectroscopy as a plausible tool with a power for the identification of not only different molecules, but their specific structures as well.

Recent years have seen both experimental progress^1^,^2^,^3^,^4^,^5^ in, as well as many theoretical proposals^6^,^7^,^8^,^9^,^10^,^11^,^12^,^13^,^14^,^15^,^16^ of, spectroscopies based on nuclear magneto-optic phenomena (termed nuclear magneto-optic spectroscopy, NMOS). NMOS methods, which manifest themselves through modulation of the polarization state of light passing through a nuclear spin-polarized sample, can be thought of as analogues of the classical magneto-optic phenomena^17^, formally obtained by exchanging the externally applied magnetic field by the field originating from a macroscopic magnetization of an ensemble of nuclear spins. In this way, Faraday rotation^18^ becomes nuclear spin-induced optical rotation (NSOR)^1^, the classical Cotton-Mouton effect^19^,^20^ becomes nuclear spin-induced Cotton-Mouton effect (NSCM)^6^,^9^,^13^,^14^, or nuclear quadrupole-induced Cotton-Mouton effect (NQCM)^12^,^15^, and the classical magnetic circular dichroism (MCD)^21^,^22^,^23^ gives rise to the nuclear spin-induced circular dichroism (NSCD)^16^.

The NMOS effects offer possibilities to develop new optical spectroscopy approaches with a high, nucleus-specific and spatial resolution, due to the fact that hyperfine interactions localized at magnetic nuclei are involved, in analogy to nuclear magnetic resonance (NMR) spectroscopy^24^. In addition, owing to the optical detection, NMOS has the potential to offer increased sensitivity and, in extensions to imaging, better resolution as compared to the relatively insensitive NMR method. Moreover, the NMO effects are based on observables differing from those of NMR. Hence, NMOS can also provide new insight into the physical properties of molecules and materials.

The first observed NMO phenomenon, NSOR, *i.e*., the rotation of linearly polarized light by an ensemble of spin-polarized nuclei, was described in 2006 by Savukov, Lee and Romalis^1^. NSOR is caused by the nuclear spin-induced antisymmetric polarizability^6^,^25^,^26^ of the molecules and is expected to feature an enhanced signal at light wavelengths close to optical transitions. Furthermore, the excited state in question may be localized to a chromophore containing the probed nucleus, thereby realizing a high spatial resolution^7^. NSOR was also predicted^7^ to feature an optical chemical shift, expressing different rotation between different molecules, as well as resolving specifically polarized inequivalent nuclear sites within one molecule. The differences in rotation between molecules have already been experimentally verified^5^, while high-resolution nuclear site specificity still awaits experimental confirmation. While NSOR is the only experimentally observed NMOS effect to date, its discovery has inspired a number of theoretical works suggesting existence of further effects^6^,^9^,^12^,^13^,^14^,^15^,^16^, among them the NSCD, the focus of this paper.

NSCD manifests itself as the difference in the absorption of left- and right-circularly polarized light or, equivalently, as ellipticity induced into the incident, linearly polarized light, in samples with macroscopic nuclear spin polarization in the direction parallel with the light beam. It should be stressed that the NSCD effect is not a perturbation of natural or magnetic circular dichroism, but has a different physical origin. NSCD arises from the real part of the nuclear spin-inflicted antisymmetric polarizability, which gains non-vanishing values only at frequencies in the vicinity of optical transitions^16^. This is in contrast to NSOR, which arises from the corresponding imaginary part, and which can be non-zero at any finite frequency. It may be speculated that NSCD will be the method of choice over NSOR in spectroscopic investigations of molecules by the analogy of NSCD with natural and magnetic circular dichroism, which are much more popular methods than the corresponding birefringent effects of optical rotation and Faraday rotation. Both the magnitude of the NSCD effect, its likely information-richer nature and the ability to single out a particular molecular species in the mixture - as it is, unlike NSOR, intimately dependent also on the specific excited state in question - underlie this anticipation.

Theoretical studies of NSCD were so far mainly concerned with small model organic molecules^16^ or, more recently, with fullerenes^27^. While it has been shown in the case of fullerenes that NSCD is capable of distinguishing different atom types within a molecule, a detailed study of the underlying effects giving rise to and influencing the shape of NSCD spectra has not yet been performed.

This paper thus dives deeper into the effects underlying NSCD spectra in order to further the understanding of this phenomenon. We show that NSCD is an effect with pronounced sensitivity to the electronic structure and that even changing the structure to a different conformer may significantly influence the spectra. This sensitivity can be linked to the changes in the electron density upon excitation. Conversely, even molecules with rather different structures can give rise to similar spectra if the spatial distribution of their excited state densities is comparable. By using a sum-over-states approach we also show that the NSCD signal can be decomposed into two contributions, which we call *B*_*d*_ and *B*_*a*_ terms based on their band-shapes resembling those characteristic for dispersion and absorption, respectively. We describe how the spectra exhibit the local nature of NSCD via the mutual orientation of the transition moments involving the orbital hyperfine operator 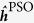 and electric dipole operator 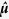. This situation is easily observable in the case of *B*_*d*_-term dominated spectra.

## Results

**Figure 1:**
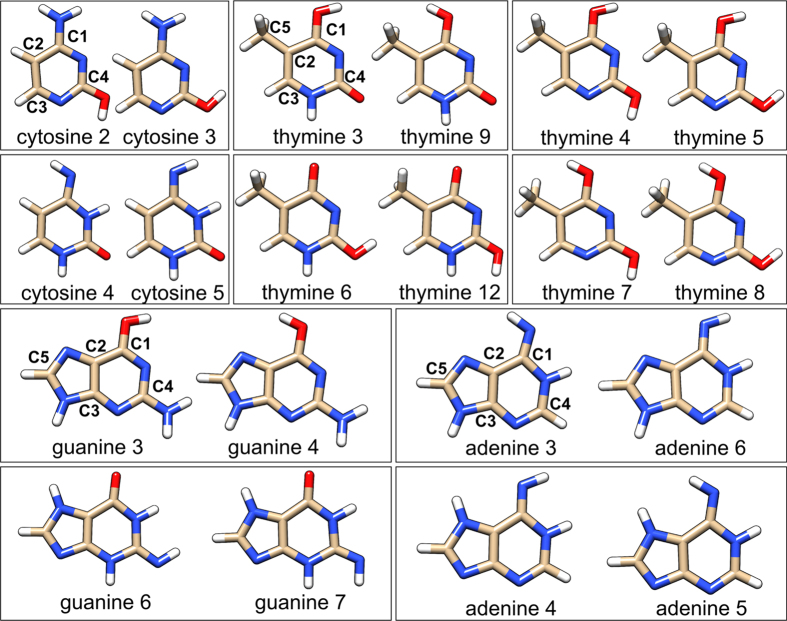
Structures of the studied four amino acid bases and their atom numbering. Numbering of the bases is taken from literature^28^.

The studied structures of all four nucleic acid bases are presented in Fig. 1. They are numbered as in the original study they were taken from^28^ and are grouped according to their structural similarity. In particular, the most similar structures, consisting of conformers corresponding to different orientations of hydroxyl or deprotonated amino groups, will be discussed together as subgroups. We will focus on cytosine and thymine in the main text and draw conclusions from there. Additional results for adenine and guanine supporting our conclusions are described in Supplementary Information (Supplementary Figs S1 and S2).

### Cytosine

**Figure 2:**
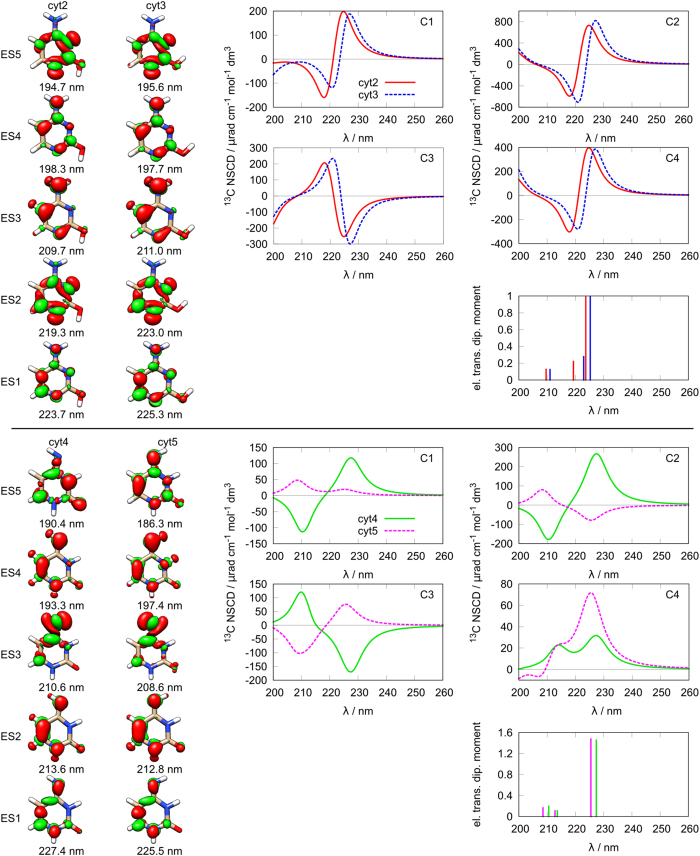
Structures and spectra of cytosine: top cyt2/cyt3; bottom: cyt4/cyt5; Left: difference densities for the first five excited states (ES 1–5); right: NSCD spectra for different carbon nuclei and electric transition dipole moment intensities.

We begin with the discussion of the NSCD spectra of the pair **cyt2**/**cyt3** shown in Fig. 2 (top) together with the difference densities corresponding to the changes of the electron cloud upon excitation from the electronic ground state to each excited state in question. As can be seen, the **cyt2**/**cyt3** conformers have remarkably similar NSCD signal as well as very similar difference densities, as could be expected since the hydroxyl group does not participate extensively in the excitations. The spectra are simple and all atoms give rise to a similar couplet feature with the two lobes at the energies of the first two excitations. The signals corresponding to the C_1_, C_2_ and C_4_ nuclei have the positive sign on the low energy side of the couplet, whereas the opposite situation pertains for the C_3_ nucleus. The reason for this behaviour lies in the different orientation of the 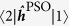 transition matrix elements for the atom C_3_, as compared to others, as will be discussed later.

In contrast to **cyt2**/**cyt3**, the pair **cyt4**/**cyt5** (Fig. 2, bottom) show marked differerences in their NSCD spectra. In the case of **cyt4**, the C_1_, C_2_ and C_3_ nuclei give rise to spectra superficially resembling those of **cyt2** and **cyt3**. However, as can be seen by comparing the corresponding excitation energies, the strong higher-energy component of the signal comes from the third excited state rather than the second one, as was the case for **cyt2**/**cyt3**. The C_4_ nucleus also behaves very differently, having mostly positive sign and lacking the couplet structure.

The spectra of the **cyt5** structure also significantly differ from those of **cyt2** and **cyt3** as well as from that of the structurally similar **cyt4**. The C_1_ and C_4_ nuclei do not reveal any couplet structure and in the case of C_2_ and C_3_ the sign pattern of the couplet is reversed as compared to **cyt4**.

The comparison of the difference densities of **cyt4** and **cyt5** shows similarity between the first two excited states, but in the third excitation the deprotonated amino group significantly influences the distribution of the density. Moreover, no clear correspondence can be found between the difference densities of **cyt4**/**cyt5** and those of **cyt2**/**cyt3** pair. These observations suggest a relation between the shape of the electron density and the NSCD spectra.

### Thymine

**Figure 3:**
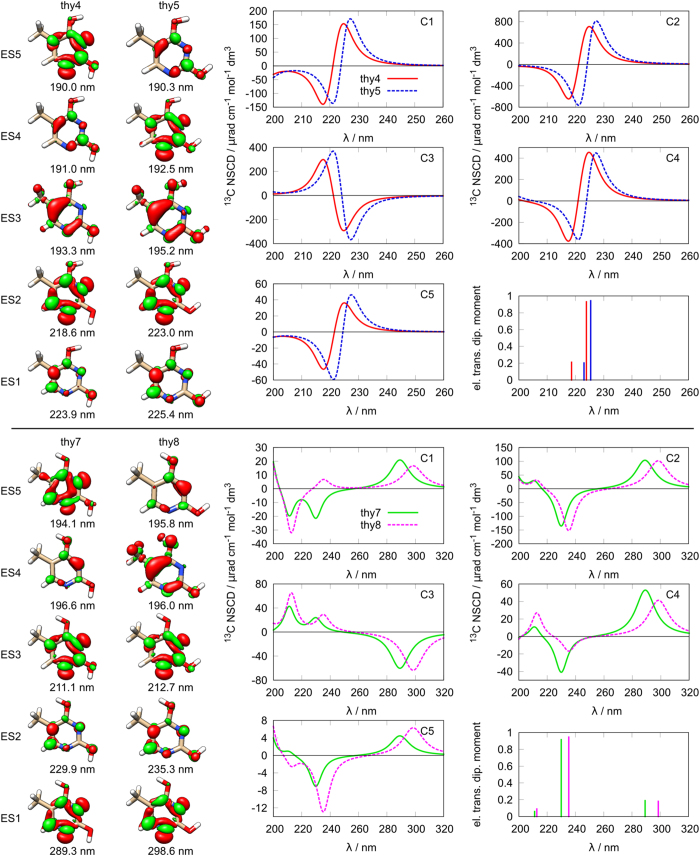
Structures and spectra of thymine: top thy4/thy5; bottom: thy7/thy8; left: difference densities for the first five excited states; right: NSCD spectra for different carbon nuclei and electric transition dipole moment intensities.

For thymine we can identify three groups of chemically similar structures **thy4**/**thy5**/**thy7**/**thy8**, **thy3**/**thy9** and **thy6**/**thy12** (Fig. 1). The first group features four different orientation combinations of the two hydroxyl groups (Fig. 3). At first glance it can be seen that, while structurally very similar, there are two pairs of molecules giving rise to two distinct sets of spectra. The first pair consists of **thy4** and **thy5** and the other pair is **thy7** and **thy8**.

The NSCD spectra of **thy4** and **thy5** (Fig. 3, top) are dominated by a couplet feature with the two lobes positioned at the first and second excited state. The carbon nuclei C_1_, C_2_, C_4_ and C_5_ produce a positive lobe on the lower energy side, while C_3_ corresponds to a negative one. Note that the intensity of the NSCD signal of the C_5_ nucleus is several times smaller than for the other nuclei, probably because of its location outside the aromatic chromophore system.

On the other hand, the **thy7**/**thy8** pair features an order-of-magnitude weaker spectra, which are, furthermore, mostly composed of isolated bands due to the wider energy separation of the excited states, as compared to the couplet-dominated case of **thy4**/**thy5**. Even though the excited-state energies are quite different from those of the **thy4**/**thy5** pair, the overall sign pattern of the spectra follows a similar trend as in the first pair, with C_3_ switching sign with respect to all the other nuclei, for the first two excited states. The only exception here is the unexpected behaviour of the C_1_ nucleus of **thy8**.

The difference densities for the structures within each of the pairs **thy4**/**thy5** and **thy7**/**thy8** are similar up to the third excited state. In fact, the densities are rather similar among all of the four structures, but their energy order is different. For example, the change in density corresponding to the first excited state in **thy4** is very similar to that for the first excited state in **thy5**, but resembles those of the second excited state for **thy7** and **thy8**.

Interestingly, a striking similarity can be observed when comparing the spectra of the **thy4**/**thy5** pair to those of **cyt2**/**cyt3**. The overall shape, position and even the intensity of the NSCD signal for carbon nuclei C_1_–C_4_ are almost identical. This appears to be somewhat more than just a fortuitous occurrence, since a comparison of the corresponding difference densities reveals a large similarity among these four structures as well. While the distributions are slightly different near the nitrogen and oxygen nuclei, their overall shapes are very similar for the first two states, which correspond to the strong couplet feature. This suggests that even structurally different molecules can give rise to similar NSCD as long as their electronically excited states are quite similar.

**Figure 4:**
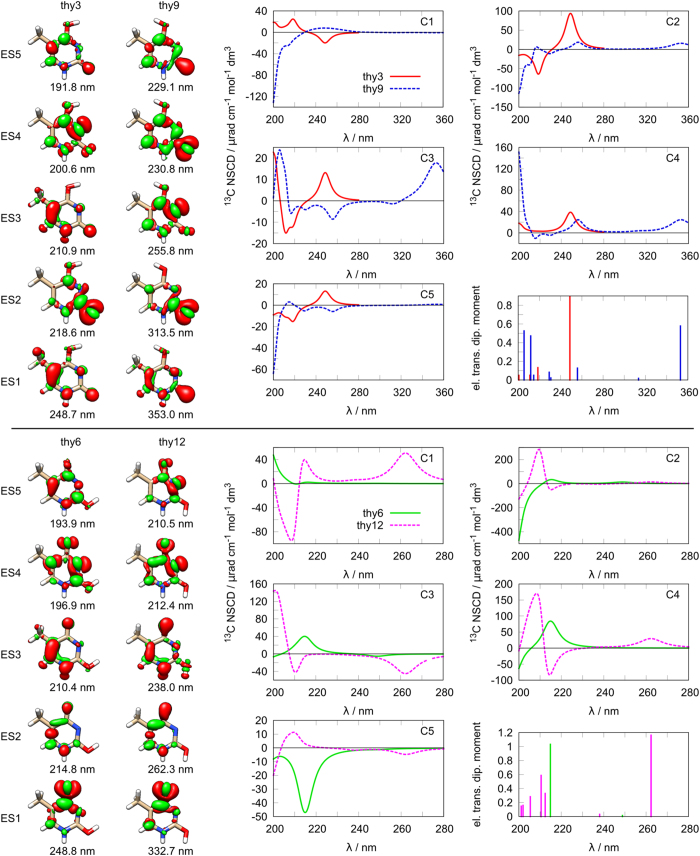
Structures and spectra of thymine: top thy3/thy9; bottom: thy6/thy12; left: difference densities for the first five excited states; right: NSCD spectra for different carbon nuclei and electric transition dipole moment intensities.

The second group of thymine structures (**thy3**/**thy9**) is shown in Fig. 4 (top). It is apparent that the spectra do not share many common features. The excited state energies are very different and no nucleus shows even a similar spectral pattern. The overall dissimilarity of the spectra is also reflected in the rather different difference densities in the pair.

The third group (Fig. 4, bottom) consists of **thy6** and **thy12**. Similarly to the previous pair, the excitation energies and the NSCD signals of these two structures are very different and no strong systematicity among the two sets can be found. It should also be noted that the difference densities of these two structures are quite different, further suggesting a rather prominent role of the nature of the excited states in NSCD, and only a minor influence of the rather similar molecular structure.

Finally, it should be also noted that no strong overall similarity can be observed between the NSCD signals of molecules from different groups of thymine structures, correlating with their dissimilar difference densities.

### ^1^H NSCD of nucleobases

The discussion so far concerned only the NSCD of ^13^C nuclei. This was done in order to demonstrate the NSCD for atoms that do not change their bonding situation between different structures - all carbon atoms are still connected to the same chemical neighbourhood in all structures of a particular base.

There is, however, another set of nuclei suitable for experiments, protons. The analysis in the ^1^H case is complicated by the fact that they migrate to various bonding sites in the different tautomers. Therefore, it is not possible to construct a one-to-one comparison of the different structures similar to that of ^13^C above, because of the fact that certain kinds of hydrogen nuclei do not exist in other structures (*e.g*., hydroxyl hydrogen absence in **cyt4** and **cyt5**). As a result, nucleus in one structure may not have the corresponding, chemically similar partner in the other one, making the comparison qualitatively different from the case of ^13^C. For this reason, the discussion of ^1^H nuclei in the present work is limited only to comparison within pairs, which consist of conformers with identical bonding arrangements. The results are included in the Supplementary Figs S3–S8 and show trends similar to the case of carbon nuclei, *i.e*., that similarities in the difference densities correspond to similar NSCD.

### Analysis of NSCD

To rationalize the NSCD signals of different nuclei, we performed an analysis in terms of the individual excited states. As the complex polarization propagator (CPP, see the Methods section) calculations used to produce the spectra of the previous figures do not allow to simply distinguish such individual contributions, we used the sum-over-states (SOS) approach, instead.

The NSCD signal is computed through quadratic response functions and can be expressed, for nucleus *K*, as^16^


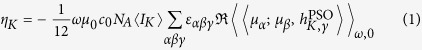


where *ω, μ*_0_, *c*_0_, *N*_*A*_ and 〈*I*_*K*_〉 refer to the angular frequency, permittivity of vacuum, speed of the light in vacuum, Avogadro constant and nuclear spin polarization, respectively. For a particular transition from the ground electronic state |0〉 to the excited state |*m*〉, NSCD is proportional to the residues of the quadratic response function^29^


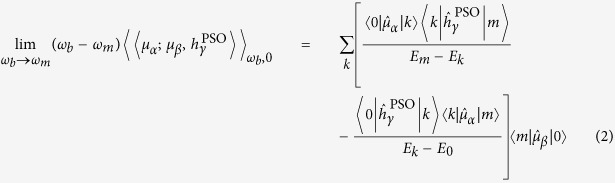


where |*k*〉 is a member of a set of further excited states with the corresponding energies of the states *E*_0_, *E*_*m*_ and *E*_*k*_, 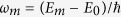 is the angular frequency corresponding to the excitation to |*m*〉, *ω*_*b*_ is the frequency of the external perturbing field, 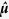 and 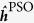 are the electric dipole and orbital magnetic hyperfine operators, respectively, and *α, β, γ* denote Cartesian coordinates.

The two contributions in eq. (2) are qualitatively different. In the first one, which we call the *B*_*d*_-term (as it appears as a dispersion bandshape, see below), both electric dipole transition moments are between the ground state and an excited state, while the matrix element of 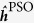 is between two excited states. In addition, the denominator of the *B*_*d*_-term contains the difference of the energies of the two excited states.

The second term in eq. (2), which we denote as the *B*_*a*_-term (since its bandshape resembles an absorption band), involves the ground state in one matrix element of the electric dipole moment, as well as in that of the 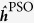 operator, whereas the transition moment between the two excited states |*m*〉 and |*k*〉 involves now the operator 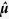. In addition, the denominator is the energy difference between the ground and the excited states in this case.

The energy denominators play a significant role in the overall NSCD intensity and the relative significance of the *B*_*d*_ and *B*_*a*_ contributions. The denominator in *B*_*a*_-type terms corresponds to excitation energies, hence it can never be smaller than the relative energy of the first excited state. In addition, the *B*_*a*_-type terms can be expected to contribute on account of the denominators progressively less to the NSCD spectrum as excited state energies rise. On the other hand, the energy difference of the two excited states in the *B*_*d*_-type terms can in principle be very small, allowing a very large contribution from this type of terms. For this reason, it may be reasonably expected that the *B*_*d*_-terms are dominant in cases of excited states packed closely together.

Another interesting feature is related to the way the *B*_*d*_ and *B*_*a*_ terms of individual transitions from the ground state contribute to the overall spectrum in eq. (1). Due to alternating Levi-Civita tensor, the imaginary 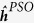 operator, and the energy difference in the denominator, the *B*_*d*_ contributions to the NSCD signal of a pair of excited states |*k*〉 and |*m*〉 are identical in magnitude but of opposite signs when the states are interchanged. Where the electric dipole operators appear “symmetrically” in the first term of eq. (2), the transition moments of the 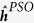 operator are between two excited states, and will change sign upon exchanging the two states. The same will occur with the energy denominator. Because of this and the properties of Levi-Civita tensor, the *B*_*d*_ terms of a pair of excited states, close to one another in energy, contribute to the spectrum (eq. (1)) with a couplet-like feature, with the two lobes of the NSCD signal at the energies of the states |*k*〉 and |*m*〉. No such symmetry exists for the *B*_*a*_ terms, so no general conclusions on the spectral shape can be easily drawn for them. Note, however, that both described terms are arising from the same perturbation of the electronic states by the local magnetic field and are physically analogous to the *B*-term in MCD. There is no *A* term-like contribution in our systems as the molecules do not possess the symmetry required for presence of degenerate states.

In order to gain further qualitative insight into the NSCD signal, we calculated NSCD spectra using the SOS expansion of eqs (1) and (2), with {|*k*〉} running over the first 10 excited states. To compare the results of the SOS procedure with those of CPP, we have numerically fitted Lorentzian lineshapes centered at each excitation energy to the CPP spectral points. The intensities obtained correlate well with the SOS results (see Supplementary Figs S9 and S10). The correlation is particularly strong when an intense *B*_*d*_ term is present due to closely spaced electronic excited states. On the other hand, weak spectra composed of mainly *B*_*a*_ terms are described less accurately by SOS with only 10 excited states included in the summation.

In the case of strong *B*_*d*_-term dominated spectra with a large magnitude of the NSCD signal, we can discuss in more detail the information content of the NSCD spectrum for such systems. As a test case we have chosen **cyt2**, as it provides a simple spectrum dominated by just one couplet, for all the ^13^C nuclei. For this case we can neglect the *B*_*a*_-term contribution altogether and approximate the *B*_*d*_ terms by including just the two interacting states - the first and the second one in eq. (2):


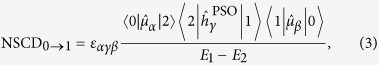



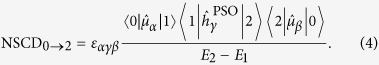


**Figure 5:**
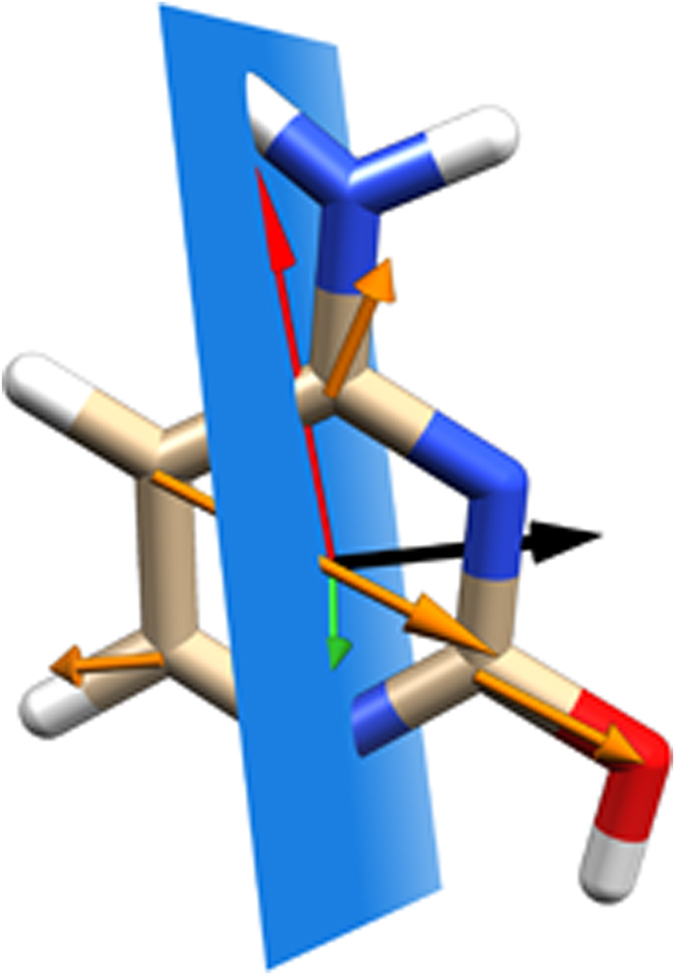
Directions of the matrix elements in cyt2: electric transition dipole moments 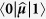 (red) 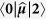 (green), the plane defined by them (blue), their cross product (black) and matrix elements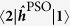 for four carbon atoms (orange).

Due to the symmetry discussed above, only eq. (3) will be discussed in the following as eq. (4) yields exactly the same numerical results, just with the opposite sign. The properties of the Levi-Civita symbol allow eq. (3) to be rewritten as


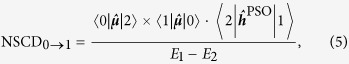


showing that the NSCD signal contains information about the magnitude of the matrix element 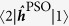 of the orbital hyperfine operator between the two excited states, as well as its direction with respect to the cross-product of electric dipole transition moments between the ground state and the excited states |1〉 and |2〉. Eq. (5) offers a deeper insight into the appearance of the NSCD spectra of the individual nuclei for **cyt2**. Since the cross-product 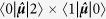 is always nucleus-independent, the only parameters modulating the appearance of the NSCD signal for different nuclei are the direction and magnitude of 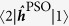. Judging by the features of the spectra of **cyt2**, it can be deduced that the projection of 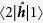 onto the product ***μ***_02_ × ***μ***_10_ for nucleus C_4_ is anti-parallel to the projection of the other three carbon nuclei. Figure 5 shows the calculated matrix elements of 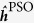 for the different carbon nuclei of **cyt2**, revealing that this is indeed the case. Hence, in the *B*_*d*_-term dominated spectra, NSCD can provide a direct measure of the component of the transition moment of the orbital hyperfine operator 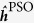 perpendicular to the plane defined by the two electric dipole transition moments involved.

Some interesting cases are particularly well-suited for analysis of NSCD in terms of the transition moments (matrix elements) of 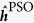 discussed above. For example, the excitation spectra of porphyrins in the Soret or Q-band region are often described in terms of two in-plane polarized orthogonal transitions^30^,^31^,^32^. For such system, NSCD would directly probe the component of matrix element of 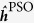 between these states perpendicular to the porphyrin ring.

## Discussion

In this paper we have performed a computational study, using a recent implementation of the complex polarization propagator-quadratic response theory method, of the theoretically predicted, but not yet experimentally observed nuclear spin-induced circular dichroism effect. Via NSCD, a sensitive optical spectroscopy with atom-specific resolution might be realized, through the modulation of the dynamic dipolar polarizability of the molecule by magnetic hyperfine interaction localized at the nuclei. We have demonstrated on a set of 20 structures of nucleic acid bases that different molecular structures of related systems provide distinct NSCD features. In contrast to NMR spectroscopy, which provides nucleus-specific resolution with the spectral parameters determined by the electronic ground state of the systems, the shape of the NSCD spectrum is heavily correlated to the differences in the electron density between the ground and excited states. Molecules that undergo similar changes in density upon optical excitation give rise to qualitatively similar NSCD signals, even when their molecular structure is rather different, such as in the case of certain structures of thymine and cytosine. Consequently, there is no simple relation between the local chemical neighbourhood of the nucleus and its spectral response, in contrast to NMR.

We have also performed a sum-over-states perturbational analysis of NSCD arising from the lowest excited states, in order to gain insight into the physical origins of the effect. It turns out that the NSCD signal can be understood as being composed from two qualitatively different contributions, which we name the *B*_*d*_- and *B*_*a*_-terms. The *B*_*d*_-term gives rise to couplet-like features in the spectra, which are particularly distinctive in molecules involving a pair of narrowly spaced excited states. The *B*_*d*_-term is related to the transition moment of the magnetic hyperfine operator between the two excited states, and it provides a measure of the component of this moment perpendicular to the plane defined by cross product between the two electric dipole transition moment vectors between the ground state and the two excited states. Moreover, for systems dominated by the *B*_*d*_-term, the sum-over-states procedure (with a limited range of states) provides a very good agreement with the rigorous response theory-based calculations. For the time being, an analogous physical interpretation relating the *B*_*a*_-term to a small number of readily interpreted contributions, remains elusive to us.

Due to its sensitivity to the structure of the excited states, NSCD shows potential for the identification of molecules based on their excitation properties. Moreover, direct insight into the relation between spatially extended electric dipole transition moments and the transition moment of the localized orbital magnetic hyperfine operator can be obtained in systems with narrowly spaced excited states, when NSCD is dominated by the *B*_*d*_-terms.

Finally, we would like to briefly comment on the experimental feasibility of the NSCD measurement. Our results predict the magnitude of the NSCD effect to be on the order of tens to hundreds of microradians for fully polarized nuclei. Therefore, the NSCD effect is actually orders of magnitude stronger than the already experimentally measured NSOR. It is reasonable to expect it to be also measurable using a similar instrumentation. For example, the inherent shortness of a single measurement limited by nuclear magnetization relaxation time can be compensated for by repeating the data acquisition or by using spin-locking techniques. This approach is clearly viable as evidenced in previous experiments with NSOR. Additionally, NSCD is modulated at the Larmor frequency of the nuclear precession, while other potentially interfering effects like natural circular dichroism or classical magneto-optic effects are static in nature and can thus be easily separated and filtered out. We believe that a continued development of this branch of nuclear magneto-optic spectroscopy may provide novel opportunities for the investigation of molecular structure and properties.

## Methods

A development version of the DALTON program package^33^,^34^ and the Turbomole 6.5 program^35^ have been used in the calculations. All molecular structures of the four bases, optimized at the B3LYP/def2-TZVPPD level, were taken from the study of Ovchinnikov and Sundholm^28^. The excitation and NSCD spectra in the present work were calculated in DALTON employing the BHandHLYP functional^36^,^37^ and a custom-made basis set derived from the def2-SVP^38^, by adding two diffuse Gaussian primitives to the angular momentum values corresponding to the occupied atomic orbitals in the ground state of the atoms involved (see Supplementary Information). The basis set was created based on previous experience^16^,^27^, showing that diffuse functions are necessary for this property to gain a faithful description of the interaction with light. The extended def2-SVP basis set was found to present a good compromise between the computational feasibility and accuracy for systems of the present size, when benchmarked against the correlation-consistent d-aug-cc-pCVDZ basis set used for NSCD in earlier work^16^. The BHandHLYP functional was presently used for all the calculations as its performance for the excitation energies and oscillator strengths^16^, as well as NSOR^7^ has been found satisfactory in comparison to coupled cluster results.

The NSCD spectra were calculated with DALTON using the complex polarization propagator (CPP) method, also termed the damped response function formalism^16^,^39^,^40^. CPP allows convergent response calculations even in the region of the spectrum where electronic transitions take place. The implementation makes use of the efficient damped response theory solver developed by Kauczor *et al*.^41^, involving an empirical linewidth parameter Γ. This parameter can be adjusted to reproduce the experimental bandwidth resulting from various factors such as solvent and vibrational effects. As there are no experimental NSCD data available yet, the damping parameter Γ = 1000 cm^−1^ was chosen to represent a reasonable situation for UV-VIS absorption bands in the aqueous phase. The NSCD signal was calculated in the wavelength range of 200–280 nm with the step of 2 nm, which covers the lowest optical excitation energies of the target systems and should be experimentally available to NSCD measurements in the future. For reasons of practicality of the forthcoming experiments, we were mainly interested in the NSCD response corresponding to the ^13^C (and ^1^H) nuclei as it is more difficult to maintain the macroscopic nuclear spin polarization for quadrupolar ^17^O and ^14^N nuclei due to their rapid relaxation, or the spin 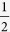 isotope ^15^N due to its low abundance.

The residues of the linear and quadratic response functions for the involved electric dipole and orbital magnetic hyperfine operators required for the sum-over-states (SOS) approach were obtained from calculation performed by the adapted DALTON code.

The excited-state differential densities were obtained using Turbomole 6.5^35^.

Molecular graphics were created using the UCSF Chimera package^42^.

## Additional Information

**How to cite this article**: Štěpánek, P. *et al*. Relation between molecular electronic structure and nuclear spin-induced circular dichroism. *Sci. Rep.* **7**, 46617; doi: 10.1038/srep46617 (2017).

**Publisher's note:** Springer Nature remains neutral with regard to jurisdictional claims in published maps and institutional affiliations.

## Supplementary Material

Supplementary Information
