# Supplementary material for: Relation between molecular electronic structure and nuclear spin-induced circular dichroism
Source: Sci Rep. 2017 Apr 24;7:46617. doi: 10.1038/srep46617 (PMC5402291; doi:10.1038/srep46617)
Supplement: Supplementary Information [file srep46617-s1.pdf]

Relation between molecular electronic structure and nuclear spin-induced circular dichroism

Petr Štěpánek, Sonia Coriani, Dage Sundholm, Vasily A. Ovchinnikov, Juha Vaara

Supporting Information

1 Results

^{13}C NSCD of guanine and adenine

This section describes ^{13}C-NSCD of guanine and adenine. Trends observed here are in agreement with the ones in the case of cytosine and thymine, described in the main text.

Guanine

The first group of guanine structures is the pair **gua3/gua4**, the spectra of which are shown in Fig. S1 (top). Strong NSCD bands can be observed in the region of the first, the third and the fourth excitations. The nucleus C_1 is quite different in this pair of structures. The first excited state gives rise to a rather strong negative signal for **gua4** while only a weak one is seen for **gua3**. On the other hand, the carbon nuclei C_2 and C_3 positioned between the two aromatic rings share common spectral features in the two structures with strong positive and negative bands at the strong first and the weaker third excitation, respectively. Likewise, nuclei C_4 and C_5 at the outer perimeter of the aromatic system of both structures also give rise to similar spectra, with positive bands at the first and the third excited states and a negative band around the fourth. The overall similarity of the NSCD signals of the two structures is again also reflected in the relative similarity of the difference densities.

The second group of guanine molecules is composed of the pair **gua6/gua7** (Fig. S1, bottom). A common feature of all the spectra is the relatively low NSCD intensity as compared to the first group of guanine structures. This is due to the different mechanism by which the NSCD spectra arise in this group, as will be discussed later. Overall, both molecules provide quite similar spectra. Nucleus C_1 features a strong negative band at the first excitation, with the other states corresponding to much smaller signals. In contrast, the signal of the C_2 carbon is strongest at the fourth excitation. C_3 is the only carbon nucleus to feature both appreciable positive and negative signals at the first and the fourth transitions, respectively. The nucleus C_4 features a strong positive band at the first excitation, whereas the other transitions are much weaker. Finally, C_5 is the only nucleus with two strong positive bands, at the first and the fourth excited state.

As can be seen, most of the spectra in this group are composed of isolated bands with no strong couplet features. In addition, all the carbon nuclei show significant differences from one another, but give similar spectra between the two molecules. The similarity of the structures is also correlated with similar difference densities.

In summary it is apparent that the NSCD spectra of group **gua3/gua4** differ from those of the pair **gua6/gua7**, but are mostly similar within each pair.

Adenine

The last presently investigated nucleobase is adenine. The first group of adenine molecules consists of the pair **ade3/ade6** with results presented in Fig. S2, (top). As can be seen, the behavior of this pair is quite complicated. The NSCD signals of nuclei C_1, C_3, C_4 and C_5 are superficially similar in both structures. For the nucleus C_1, the similarity is in the overall features, such as in the predominantly positive sign. C_3 carries a positive signal for the first two bands in both structures, followed by a negative NSCD at higher energy. C_4 features a rather strong positive band at the first excitation followed by weaker negative signal. C_5 shows first two bands as positive, followed by third and fourth excitation forming a couplet. In contrast, C_2 behaves rather differently for **ade3** and **ade6**. While the first and second excitations are positive and negative, respectively, in both structures, the higher states have opposite signs for them. The difference densities of the **ade3/ade6** pair are principally similar with modest difference observable, *e.g.*, in the second excited state.

The second pair of structures is **ade4/ade5** (Fig. S2, bottom). Overall the trends here are somewhat similar to those in the **ade3/ade6** pair. C_3, C_4 and C_5 again give the most similar spectra for the two structures. The NSCD spectrum of nucleus C_3 is positive for the first excited state, followed by negative bands. The nucleus C_4 also exhibits a couplet similar to that in the first pair of structures, between the first two excited states. C_5 continues to show a couplet between the third and the fourth excited state, with the lower-energy states corresponding to positive NSCD. In contrast, C_1 gives rise to rather different signals between **ade4** and **ade5**. Whereas **ade4** has a strong couplet feature between excited states 3 and 4, this feature is not present in **ade5**. Finally, similarly to the first group, the signals of C_2 are quite different within the **ade4/ade5** pair.

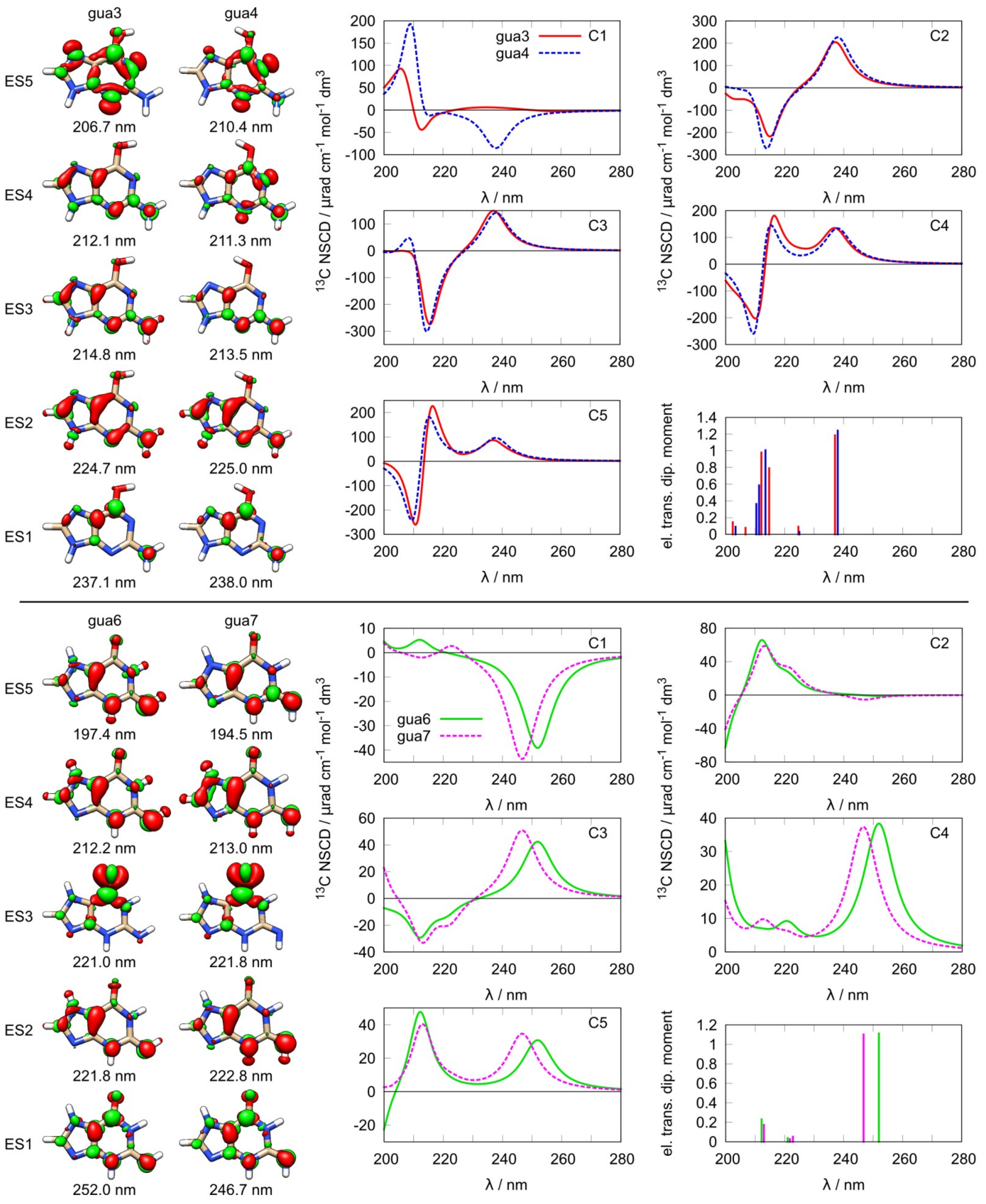

S 1. Structures and spectra of guanine: top: **gua3/gua4**; bottom: **gua6/gua7**; left: difference densities for the first five excited states; right: NSCD spectra for different carbon nuclei and electric transition dipole moment intensities.

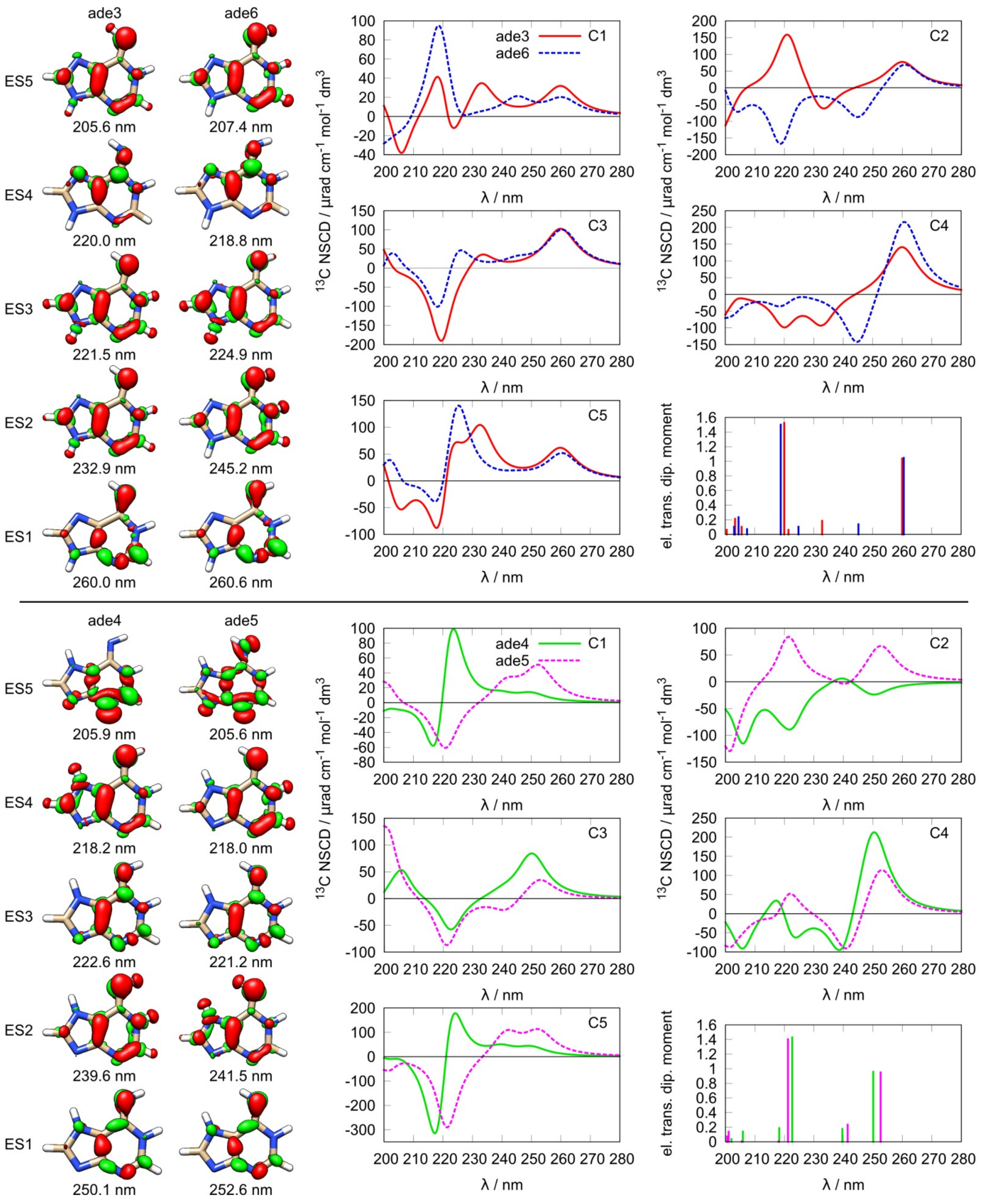

S 2. Structures and spectra of adenine: top **ade3/ade6**; bottom: **ade4/ade5**; left: difference densities for the first five excited states; right: NSCD spectra for different carbon nuclei and electric transition dipole moment intensities.

An interesting point is the similarity of the NSCD of the nucleus C_2 for both **ade6** and **ade4** and also for **ade3/ade5**. While there are clear differences in details, these pairs display for C_2 better matching NSCD response than with the partner in their own group.

In summary, the overall shape of the NSCD spectrum for both groups is rather similar. This makes adenine, among the investigated systems, the molecule with overall the most similar NSCD between its different tautomers. The degree of similarity is reflected in the somewhat similar difference densities for all the four structures of adenine, at least for the first three excited states.

^{1}H NSCD of nucleobases

This section briefly discussed the ^{1}H NSCD. The numbering of atoms is give in Fig. S3.

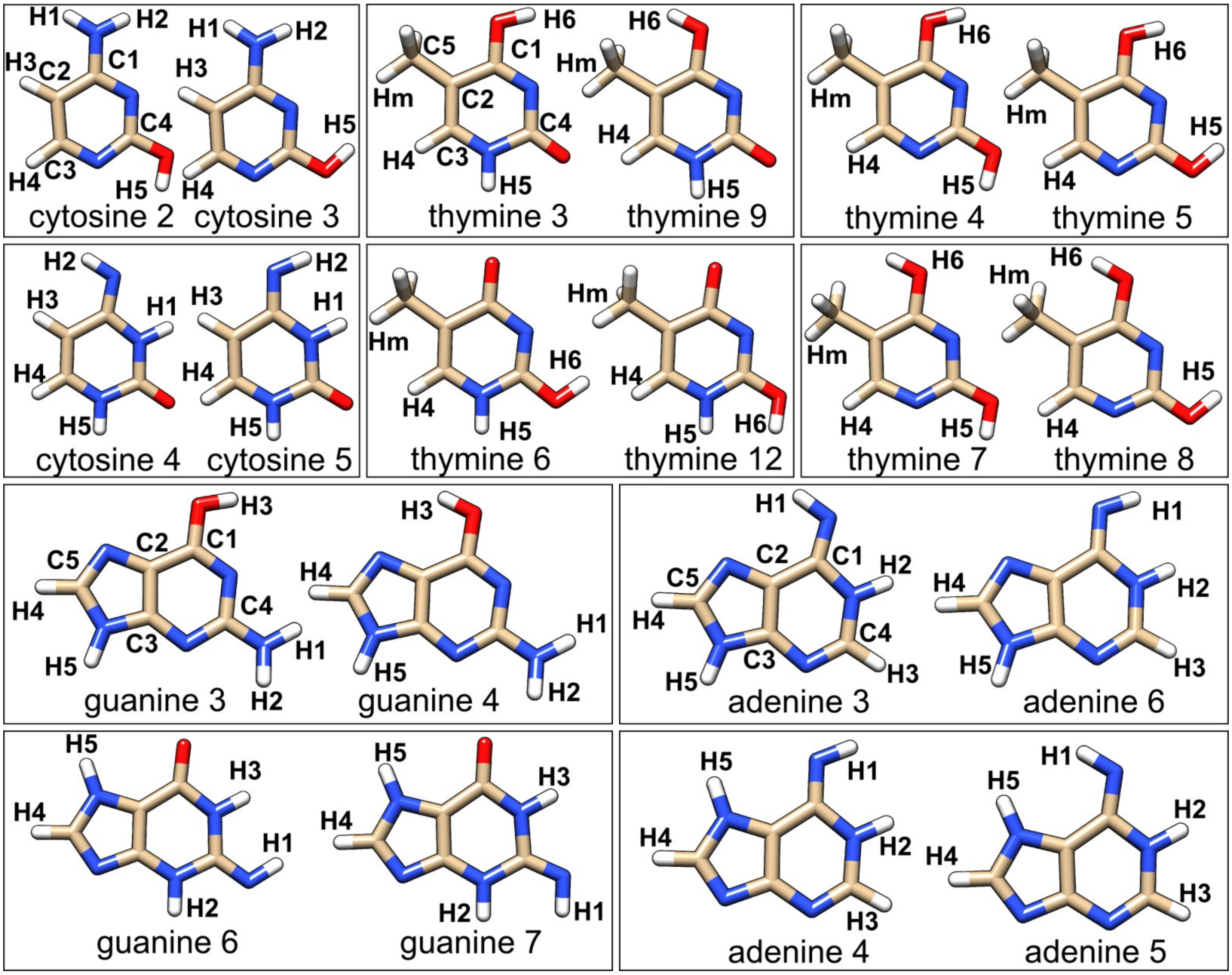

S 3. Numbering of hydrogen atoms.

1.1 Cytosine

In the case of cytosine (Fig. S4), the general trends are very similar to those for ^{13}C NSCD, namely the **cyt2/cyt3** pair show very similar spectra and the **cyt4/cyt5** exhibit significant differences.

However, there are several interesting features, especially in the case of the **cyt2/cyt3** pair. Firstly, the spectral shapes are more varied than in the case of carbon, which was composed only of simple couplets. This is especially seen in the H5. Moreover, while the hydrogen nuclei H1 and H2 retain the shape of couplets, they are no longer symmetric. These changes can be rationalized using the sum-over-state analysis, which reveals that the intensity of the first transition is not only due to the coupling with the second excited state, but also with a significant contribution from the fourth state. The larger energy

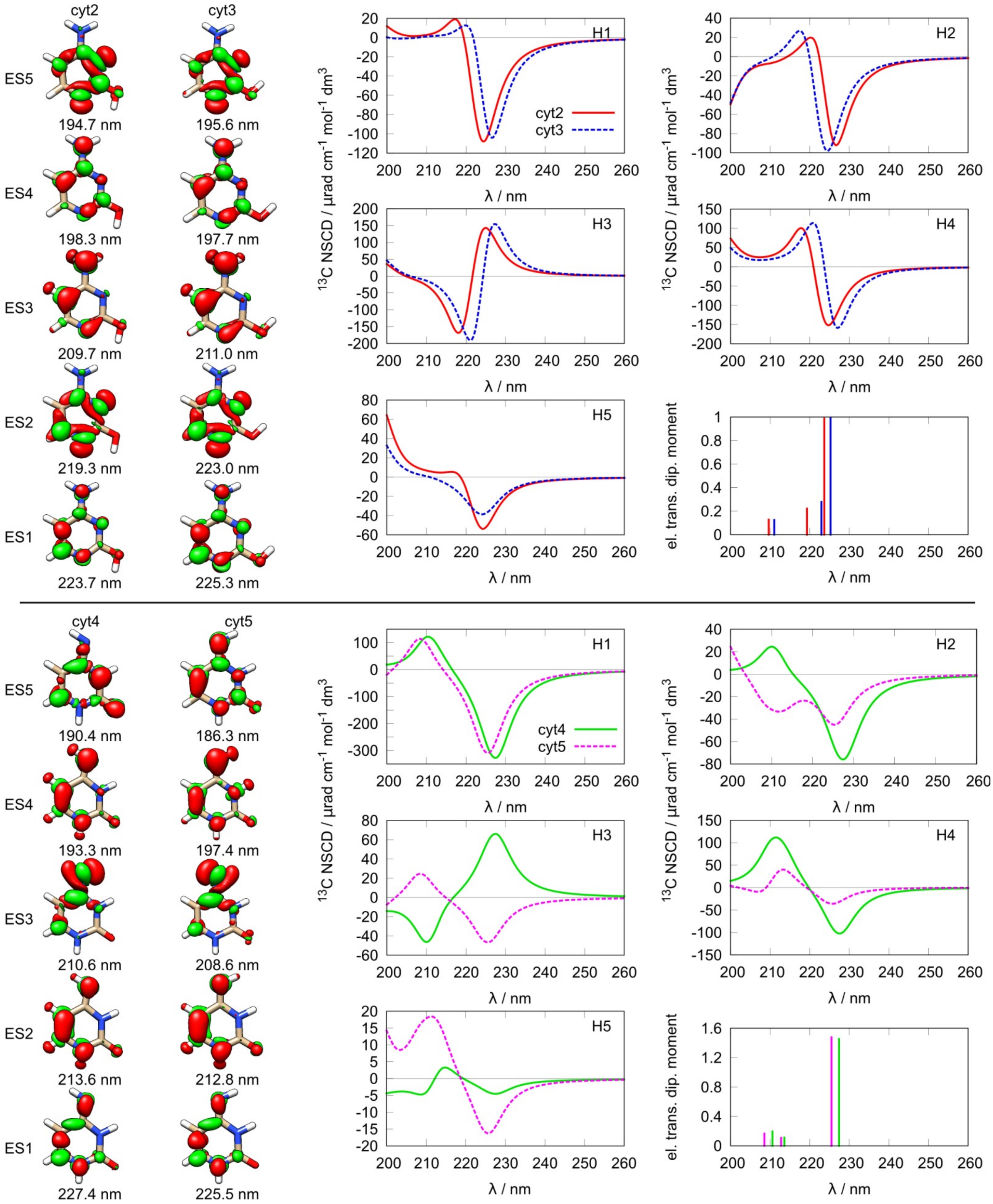

S 4. ^{1}H-NSCD spectra of cytosine structures **cyt2**, **cyt3**, **cyt4**, **cyt5**.

difference between these two states is compensated by the order of magnitude larger matrix element $\langle 1|h^{\mathrm{PSO}}|4\rangle$ than $\langle 1|h^{\mathrm{PSO}}|2\rangle$ which makes this contribution significant for hydrogens H1 and H2.

1.2 Thymine

The ^{1}H spectra of thymines **thy4**, **thy5**, **thy7** and **thy8** (Fig. S5) show behaviour very similar to the ^{13}C nuclei in being quite similar to each member of the pairs. It is worth noting that while the carbon nuclei show significant agreement of **cyt2/cyt3** with **thy4/thy5**, this agreement is not preserved in the proton NSCD. This can be attributed to the very different chemical nature of ^{1}H nuclei in cytosine and thymine.

Moreover, it is worth noting that the second excited state in the **thy7/thy8** pair also exhibit a deviation in the spectra in the case of nucleus H5, while the other spectra are rather consistent. This is similar to the case of carbon nucleus C1 in this pair.

The second subset of thymines (Fig. S6) also shows a behaviour similar to the case of carbons. Namely, the paired molecules do not show significant agreement in spectra despite their structural similarity.

1.2.1 Guanine

^{1}H NSCD spectra of guanine are shown in Fig. S7. It can be seen that the large similarity of the densities that corresponds to the carbon spectra that are in close correspondence to one another, is also manifested in the hydrogen nuclei. In fact, it can be argued that, in this case, the ^{1}H spectra are even more similar to each other than the ^{13}C spectra.

1.2.2 Adenine

The case of adenine spectra is plotted in Fig. S8. Similarly to the case of carbon, the relative correlation between similarity of the difference densities and spectra is rather well-preserved in this case, as well. That means that the general shape of the spectra is rather similar between the molecules in each pair, although a number of differences can be observed in the details.

In conclusion, the ^{1}H and ^{13}C spectra show an overall similar level of agreement between the individual molecules.

SOS approach to NSCD

The data for comparison of SOS and CPP approach were obtained in the following way:

- CPP intensities were obtained as a best numerical fit to the spectra calculated by CPP. Position of the peaks were given by excitation energies calculated on the identical level of theory as the NSCD and the width of the peaks is defined by the factor Γ in the calculations.
- SOS intensities were calculated as

$$\mathrm{NSCD}_{0\rightarrow k,K} = A\sum_{k}\left[\frac{\langle 0|\hat{\mu}_{\alpha}|k\rangle\,\langle k|\hat{h}_{\gamma}^{\mathrm{PSO}}|m\rangle}{E_m - E_k} - \frac{\langle 0|\hat{h}_{\gamma}^{\mathrm{PSO}}|k\rangle\,\langle k|\hat{\mu}_{\alpha}|m\rangle}{E_k - E_0}\right]\langle m|\hat{\mu}_{\beta}|0\rangle$$

  where prefactor A is defined as

$$A = \gamma_K I_K \frac{c_0 N_A e^3 \hbar}{10 E_H a_0 m_e}$$

  where c_0, N_A, e, E_H, a_0, m_e is speed of light in vacuum, Avogadro constant, elementary charge, energy of 1 Hartree in J, Bohr radius and mass of electron.

The Fig. S9 show the correlation between CPP and SOS approach. The plots include only the first three transitions $m \leftarrow 0$ for all the structures, to limit the influence of the finite linewidth of transitions higher in energy and out of the calculated spectral range (”tailing” of the bands higher in energy into the low energy regions). It can be seen that the inclusion of even as few as 10 states in the SOS procedure does indeed capture the general qualitative trends of the NSCD signals. Precautions must be taken, however, when interpreting these results. The NSCD spectrum can, for example, be dominated by only a few important large terms, a likely case due to a strong B_d-terms for a pair of close lying excited states. However, in some systems, the total signal can be expected to be reconstructed only by using a large number of small contributions, in which case the SOS procedure up to 10 states may not be sufficient for a faithful reproduction of the response theory results.

To illustrate the possible issues with the performance of the SOS method, we plot the SOS-NSCD against CPP-NSCD in the different groups of tautomers of the DNA bases, in Fig. S10. The first archetypical case, **cyt2**, is dominated by the interaction of the first and the second excited state, which lie very close in energy. The second case is represented by **gua6**, the molecule with one of the weakest NSCD responses in this study. Since **cyt2** is dominated by B_d-term interaction of the first two states, the 10-states description is sufficient. On the other hand, no two excited states strongly couple together to form a strong B_d-type couplet in the case of **gua6**, and the NSCD spectrum is in this case composed of a large number of small contributions.

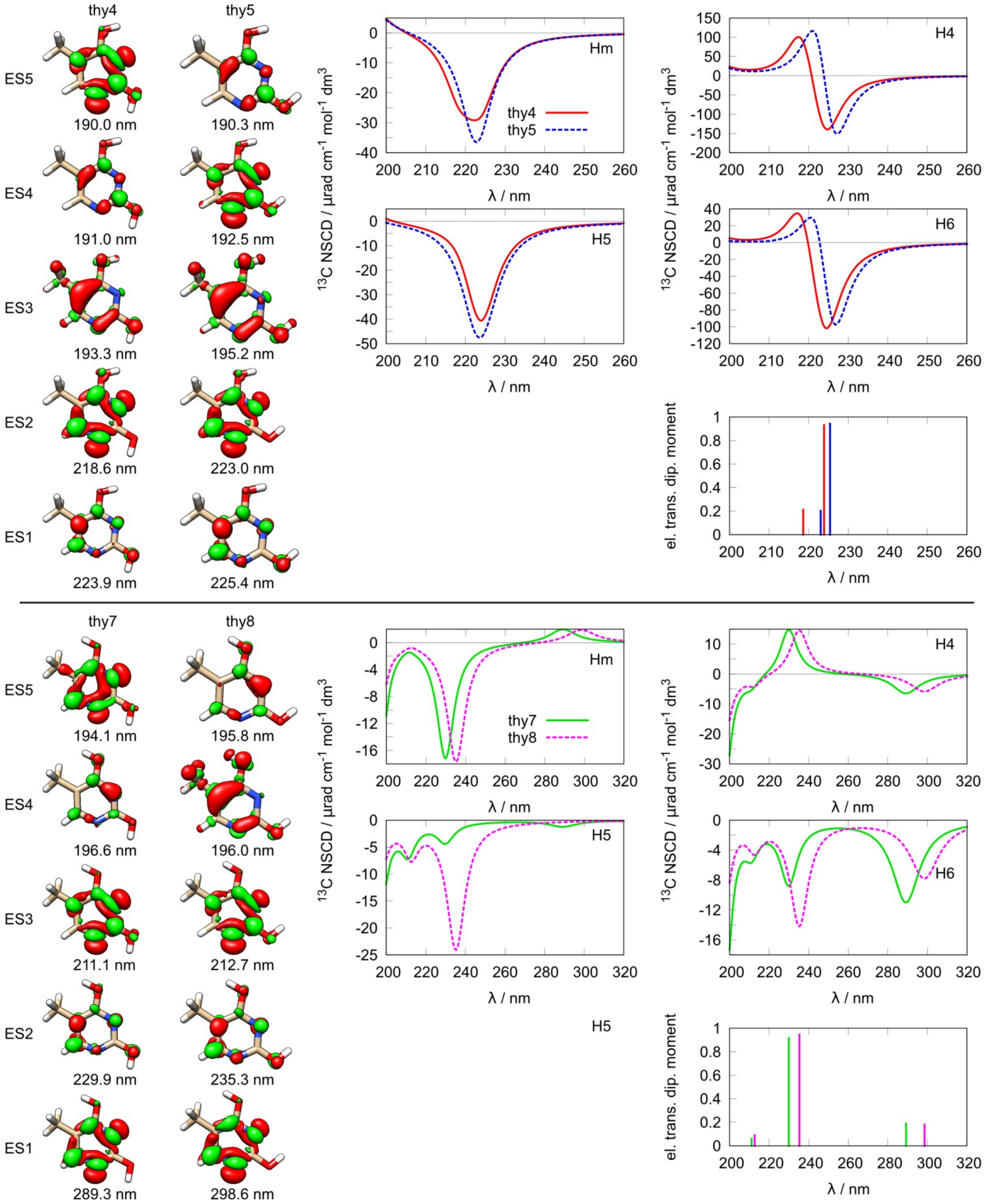

S 5. ^{1}H-NSCD spectra of thymine structures **thy4**, **thy5**,**thy7** and **thy8**.

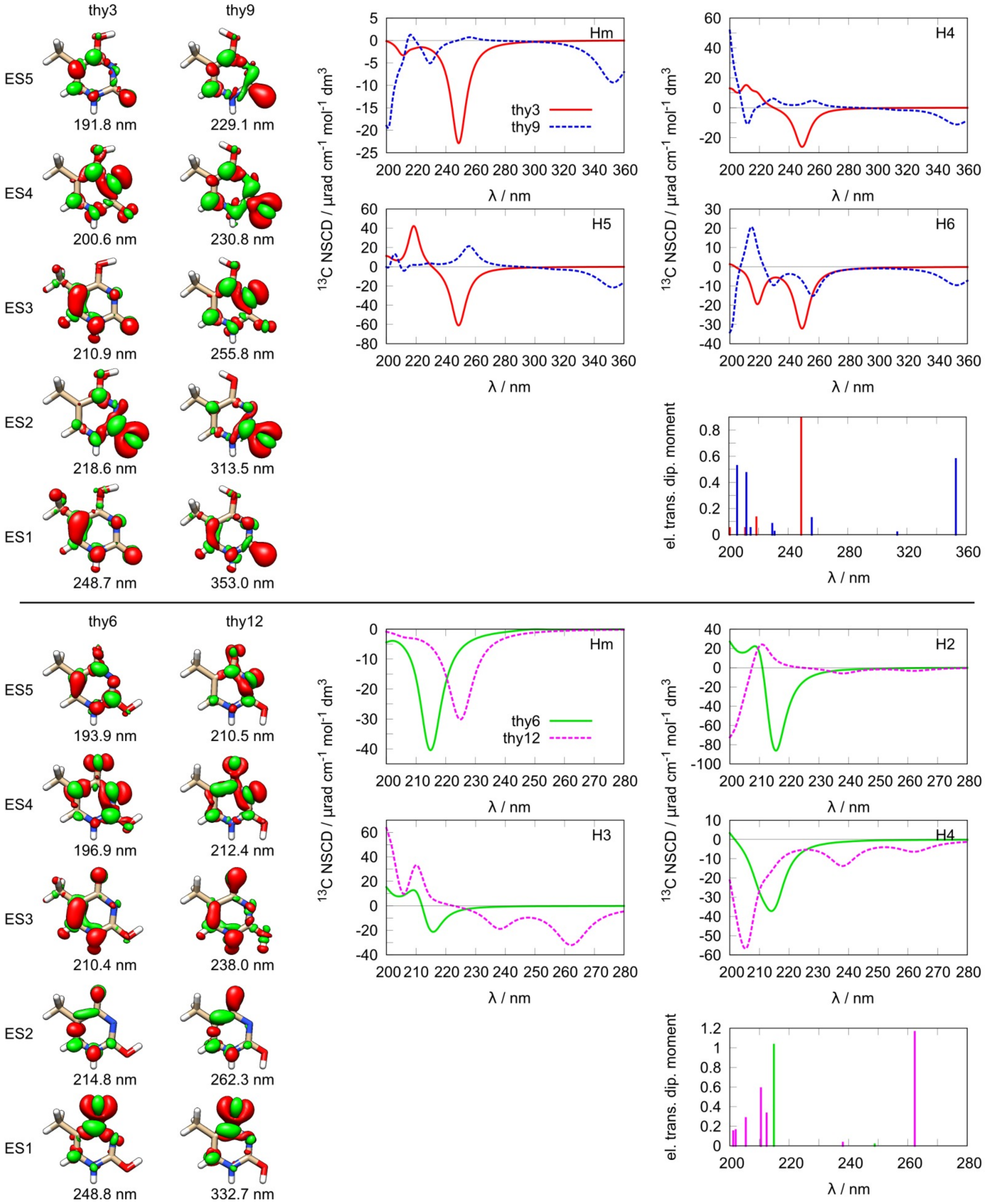

S 6. ^{1}H-NSCD spectra of thymine structures **thy3**, **thy9**, **thy6** and **thy12**.

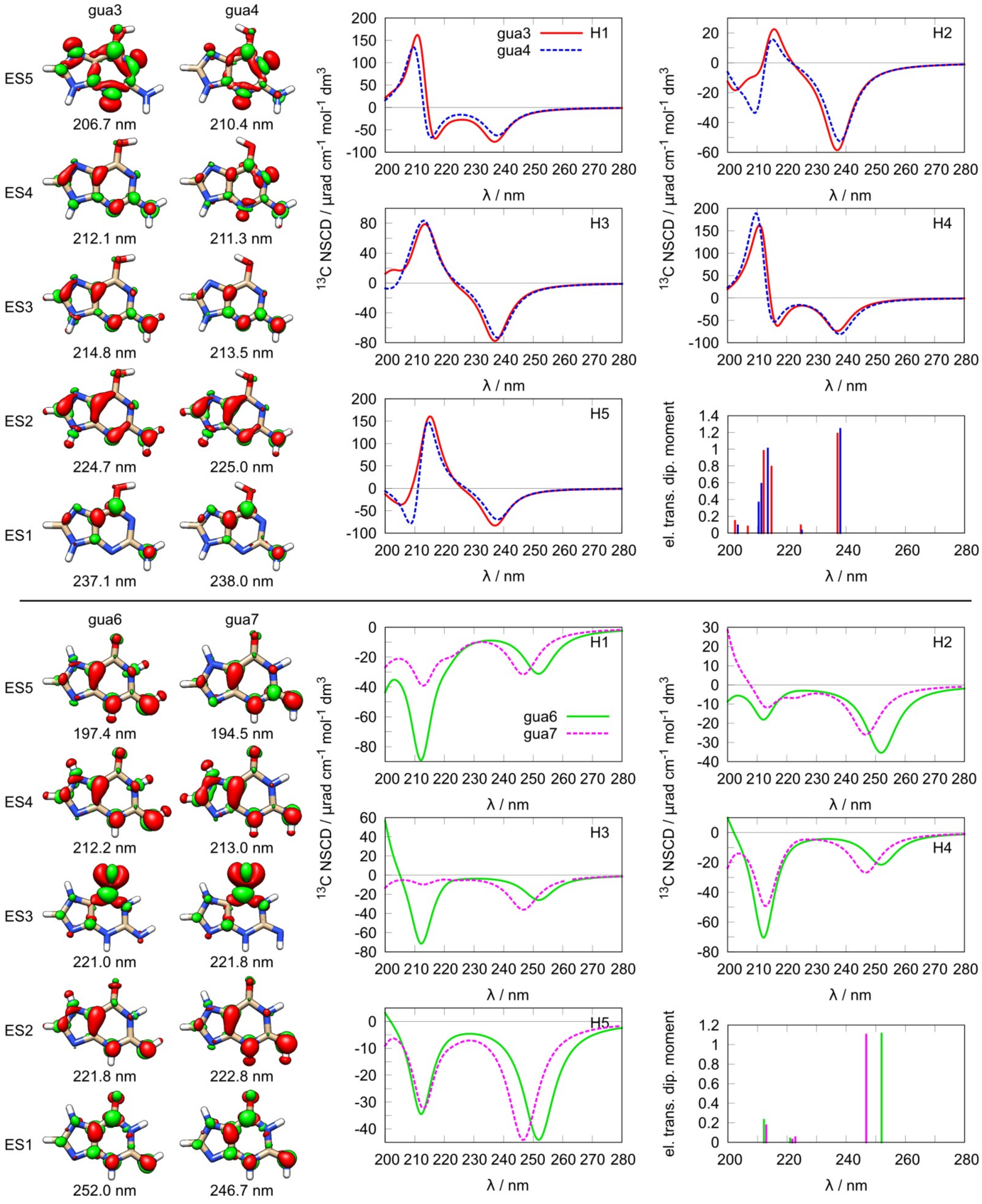

S 7. ^{1}H-NSCD spectra of guanine structures **gua3**, **gua4**, **gua6**, **gua7**.

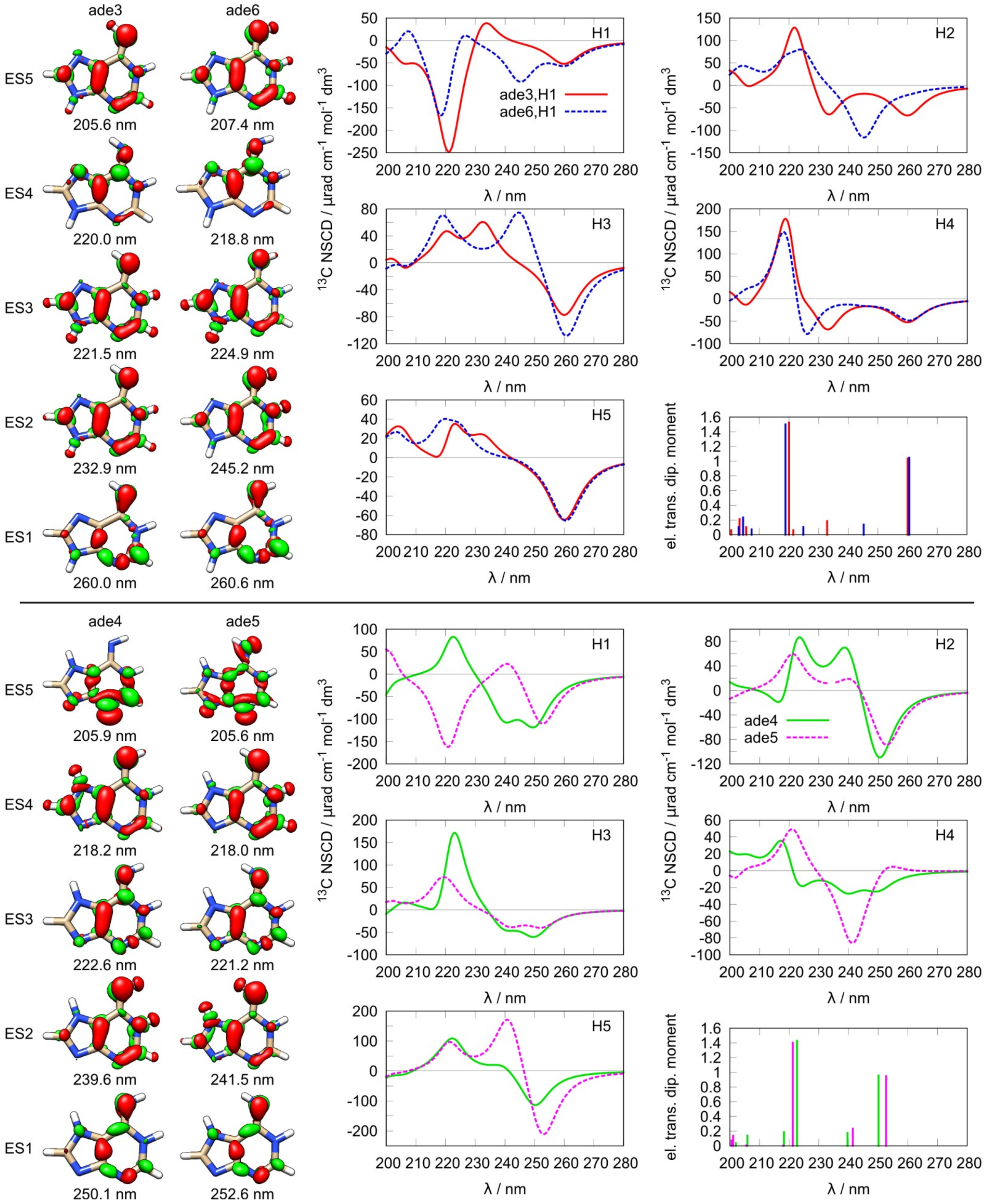

S 8. ^{1}H-NSCD spectra of adenine structures **ade3**, **ade6**, **ade4**, **ade5**.

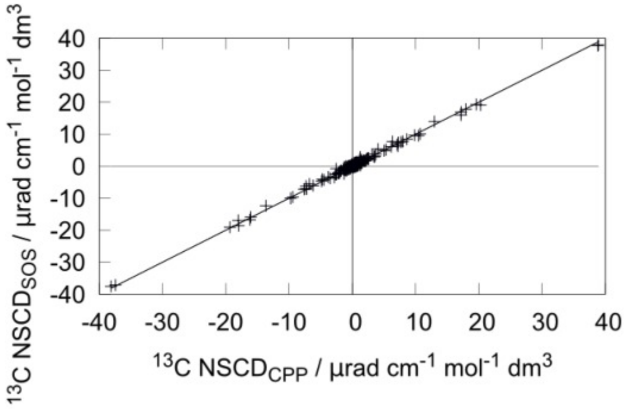

S 9. Correlation of the NSCD signals of the first three transitions for all the 20 studied structures of DNA bases obtained by the fit to the complex polarization propagator (CPP) spectra and by the sum-over-states (SOS) approach, ranging up to 10 states.

In this case the 10-state SOS description is clearly lacking. Since the most intense signals across the systems studied come from B_d-type contributions, CPP-NSCD correlates overall very well with SOS-NSCD (with a limited number of excited states accounted for) in Fig. S9, but care should be taken for the weak signals, *i.e.*, points lying close to the center of the plot.

2 Methods

The basis set used was derived from the def2-SVP basis set by augmenting the l-shells corresponding to the occupied atomic orbitals with two additional diffuse functions. The new exponents were calculated by twice consecutively dividing the lowest existing exponent by the factor of three. The resulting basis sets were as listed below

HYDROGEN

s functions

6 4 0

13.01070100	0.01968216	0.00000000	0.00000000	0.00000000
1.96225720	0.13796524	0.00000000	0.00000000	0.00000000
0.44453796	0.47831935	0.00000000	0.00000000	0.00000000
0.12194962	1.00000000			
0.04064987	1.00000000			
0.01354996	1.00000000			

p functions

1 1 0

0.80000000	1.00000000

CARBON

s functions

9 5 0

1238.40169380	0.00545688	0.00000000	0.00000000	0.00000000	0.00000000
186.29004992	0.04063841	0.00000000	0.00000000	0.00000000	0.00000000
42.25117635	0.18025594	0.00000000	0.00000000	0.00000000	0.00000000
11.67655793	0.46315122	0.00000000	0.00000000	0.00000000	0.00000000
3.59305065	0.44087173	0.00000000	0.00000000	0.00000000	0.00000000

0.40245147	1.00000000
0.13090183	1.00000000
0.04363394	1.00000000
0.01454465	1.00000000

p functions

6 4 0

9.46809706	0.03838787	0.00000000	0.00000000	0.00000000
2.01035451	0.21117025	0.00000000	0.00000000	0.00000000
0.54771005	0.51328172	0.00000000	0.00000000	0.00000000
0.15268614	1.00000000			
0.05089538	1.00000000			
0.01696512	1.00000000			

d functions

1 1 0

0.8000000	1.00000000

NITROGEN

s functions

9 5 0

1712.8415853	-0.00539341	0.00000000	0.00000000	0.00000000	0.00000000
257.64812677	-0.04022158	0.00000000	0.00000000	0.00000000	0.00000000
58.45824585	-0.17931145	0.00000000	0.00000000	0.00000000	0.00000000
16.19836791	-0.46376318	0.00000000	0.00000000	0.00000000	0.00000000
5.00526008	-0.44171423	0.00000000	0.00000000	0.00000000	0.00000000
0.58731857	1.00000000				
0.18764592	1.00000000				
0.06254864	1.00000000				
0.02084955	1.00000000				

p functions

6 4 0

13.57147023	-0.04007240	0.00000000	0.00000000	0.00000000
2.92573729	-0.21807045	0.00000000	0.00000000	0.00000000
0.79927751	-0.51294466	0.00000000	0.00000000	0.00000000
0.21954348	1.00000000			
0.07318116	1.00000000			
0.02439372	1.00000000			

d functions

1 1 0					
1.0000000	1.00000000				

OXYGEN

s functions

9 5 0					
2266.17677850	-0.00534318	0.00000000	0.00000000	0.00000000	0.00000000
340.87010191	-0.03989004	0.00000000	0.00000000	0.00000000	0.00000000
77.36313517	-0.17853912	0.00000000	0.00000000	0.00000000	0.00000000
21.47964494	-0.46427685	0.00000000	0.00000000	0.00000000	0.00000000
6.65894331	-0.44309745	0.00000000	0.00000000	0.00000000	0.00000000
0.80975976	1.00000000				
0.25530772	1.00000000				
0.08510257	1.00000000				
0.02836752	1.00000000				

p functions

6 4 0				
17.72150432	0.04339457	0.00000000	0.00000000	0.00000000
3.86355054	0.23094121	0.00000000	0.00000000	0.00000000
1.04809209	0.51375311	0.00000000	0.00000000	0.00000000
0.27641544	1.00000000			
0.09213848	1.00000000			
0.03071283	1.00000000			

d functions

1 1 0	
1.2000000	1.00000000

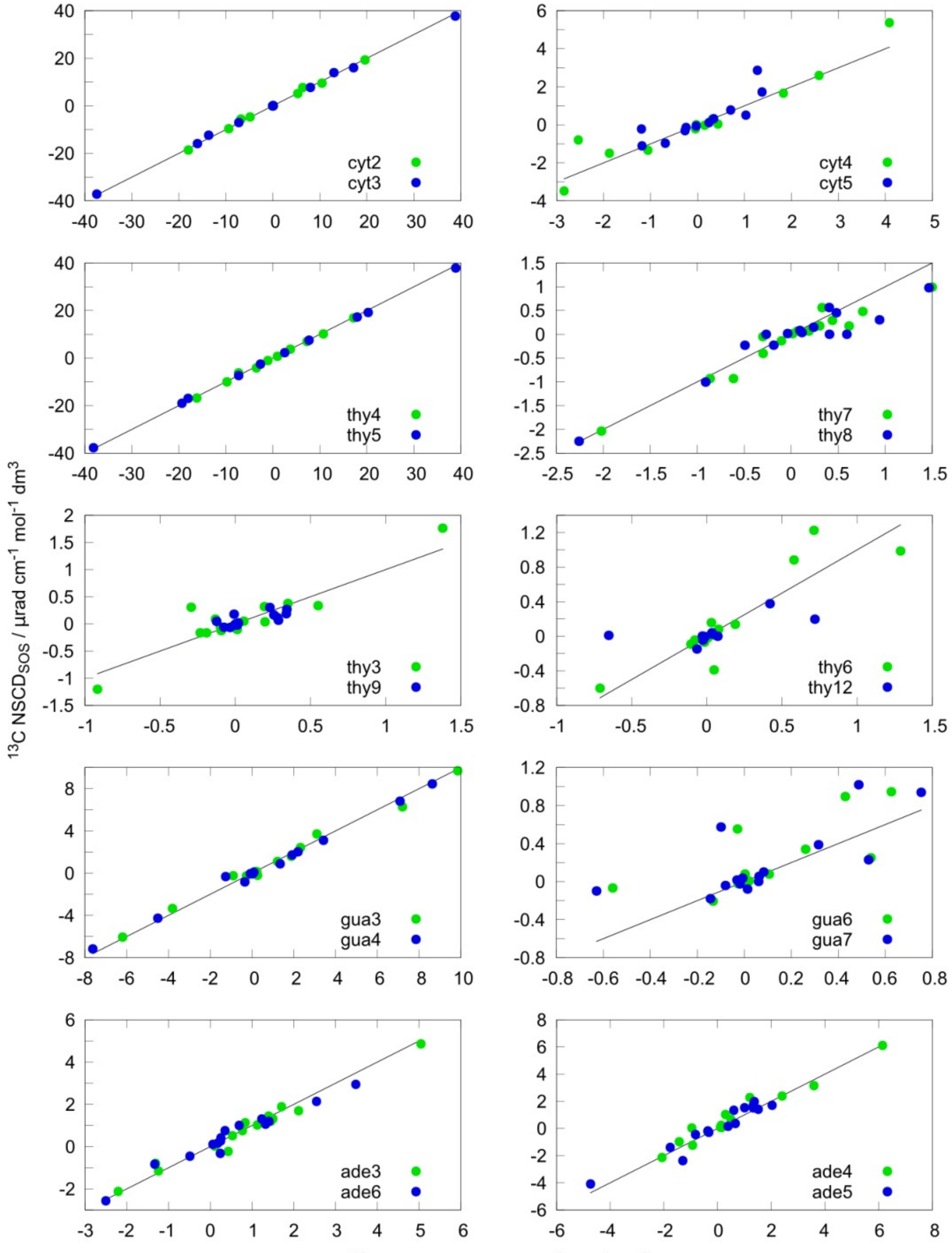

S 10. Correlation of the NSCD signals of the first three transitions for all the 20 studied structures of DNA bases obtained by the fit to the complex polarization propagator (CPP) spectra and by the sum-over-states (SOS) approach, ranging up to 10 states. Individual plots correspond to pairs discussed in the main text. A clearly stronger overall correlation can be observed in generally stronger signals.
